# Dietary Supplementation of Fruit from *Nitraria tangutorum* Improved Immunity and Abundance of Beneficial Ruminal Bacteria in Hu Sheep

**DOI:** 10.3390/ani12223211

**Published:** 2022-11-19

**Authors:** Xia Du, Xindong Cheng, Qiaoxia Dong, Jianwei Zhou, Abraham Allan Degen, Dan Jiao, Kaixi Ji, Yanping Liang, Xiukun Wu, Guo Yang

**Affiliations:** 1 Northwest Institute of Eco-Environment and Resources, Chinese Academy of Sciences, Lanzhou 730000, China; 2 University of Chinese Academy of Sciences, Beijing 100049, China; 3 Key Laboratory of Stress Physiology and Ecology, Gansu Province, Lanzhou 730000, China; 4 State Key Laboratory of Grassland and Agro-Ecosystems, College of Pastoral Agriculture Science and Technology, Lanzhou University, Lanzhou 730000, China; 5 Desert Animal Adaptations and Husbandry, Wyler Department of Dryland Agriculture, Blaustein Institutes for Desert Research, Ben-Gurion University of Negev, Beer Sheva 8410500, Israel; 6 Key Laboratory of Extreme Environmental Microbial Resources and Engineering, Lanzhou 730000, China

**Keywords:** bioactive substances, Hu rams, medicinal plants, rumen fermentation, rumen microbiota, serum biochemistry

## Abstract

**Simple Summary:**

The fruit of *Nitraria tangutorum* (FNT), a medicinal, edible plant, contains a variety of bioactive ingredients with a number of biological functions. However, its effect on sheep is unknown. To fill this gap, we examined the effects of supplementary FNT on serum biochemistry and the rumen bacteria in Hu rams. The results demonstrated that FNT could improve the immunity of sheep and increase the relative abundance of beneficial rumen bacteria. However, the rumen fermentation variables were not altered with supplementary FNT.

**Abstract:**

The fruit of *Nitraria tangutorum* (FNT) is reputed to possess medicinal properties; however, its effect on sheep (*Ovis aries*) is unknown. The aim of this study was to fill this gap. In a 3 × 3 Latin square design, six 12-month-old rumen-fistulated Hu rams (56.2 ± 8.26 kg; mean ± SD) were penned individually and offered one of three levels of FNT, namely, 0 g/d (control; CON), 16 g/d (N_16_), and 48 g/d (N_48_). The concentration of serum immunoglobulin G increased linearly (*p* = 0.03) with an increasing intake of FNT. The serum concentration of β-hydroxybutyrate in the N_48_ group was lower than in the CON group (*p* = 0.01) and decreased linearly with increasing FNT (*p* = 0.001). The concentration of serum lactate dehydrogenase tended to decrease (*p* = 0.07) linearly with an increase in FNT intake, while the concentration of glucose did not differ among groups (*p* = 0.14) but displayed a quadratic curve with an increase in FNT (*p* = 0.05). The rumen concentration of lipase decreased linearly with increasing FNT (*p* = 0.04). The rumen fermentation variables were not affected by FNT. The FNT intake increased the abundance of beneficial ruminal bacteria, such as *Lachnoclostridium*, Rhodocyclaceae, and *Candidatus* Arthromitus. *Prevotella*, *Rikenellaceae*_*RC9*_*gut*_*group*, *Ruminococcus*, *Olsenella*, *Lachnospiraceae*_*NK3A20*_*group*, and *Quinella* were the dominant bacterial genera in all treatments. We conclude that FNT can improve immunity and increase the relative abundance of beneficial ruminal bacteria in sheep.

## 1. Introduction

Chemicals and antibiotics are used widely as additives in animal feed to improve production. However, some of these additives have been banned in many countries due to negative effects on the environment and on human health [1,2]. Natural herbs are being tested as replacements for banned substances and studies have reported that some Chinese herbal plants have beneficial effects on immunology [3], growth performance [4,5], and rumen health [6,7,8] in sheep (*Ovis aries*).

*Nitraria tangutorum*, a perennial deciduous shrub belonging to the Zygophyllaceae family, is distributed widely in the deserts and semi-deserts of Asia, Europe, Africa, and Australia [9,10]. The fruit of *N. tangutorum* (FNT) contains abundant flavonoids, alkaloids, quercetin, and polysaccharides [11]. In Chinese traditional medicine, FNT has long been used to treat spleen and stomach disorders, neurasthenia, dizziness, and dyspepsia [12]. FNT is reputed to possess a variety of beneficial pharmacological properties, at least for humans and mice (*Mus musculus*). Mice displayed anti-fatigue behavior after oral intake (50 to 200 mg/kg) of an extract of FNT water-soluble polysaccharides for 15 days [13]. François et al. reported that the ethanol extract of FNT (0.1–10 g/L) promoted vasodilation and anti-hypertension in rats (*Rattus norvegicus*) [14]. Polysaccharides (10 mg/mL) from FNT acted as a protective agent on lipopolysaccharide (LPS)-induced acute lung injury in mice by inhibiting the TLR4 inflammatory signaling pathway [15], while proanthocyanins demonstrated neuroprotective effects by reducing oxidative stress, reducing the accumulation of β-amyloid protein and inhibiting the proliferation of glial cells in rats [16]. In addition, proanthocyanins extracted from the by-products of FNT showed protective effects against doxorubicin-induced H9c2 cardiomyocyte injury [17]. *Nitraria retusa* extract (300 mg/kg body weight) had a protective effect on penconazole-induced kidney injury by reducing the levels of plasma creatinine, urea, uric acid, and lactate dehydrogenase and improving kidney tissue lesions [18]. The antioxidant components of FNT (25, 50, or 100 mg/kg) reduced cerebral infarction size, improved neurological function defects, reduced pro-inflammatory factors, improved brain injury, and displayed neuroprotective effects in C57BL/6 stroke mice [19]. In addition, an extract of FNT (400 mg/kg) reduced the level of starch/maltose/sucrose-mediated postprandial blood glucose in diabetic mice [20].

FNT is used as a feed additive for livestock in some regions of China; however, to date, little is known about its effect on ruminants. The aim of this study was to fill this important knowledge gap. In this study, we examined the effects of supplementary FNT on serum metabolites, rumen fermentation variables, and rumen microbiota.

## 2. Materials and Methods

The experimental procedures, animal handling, and sample collections were approved by the Animal Welfare and Experimental Ethics Committee of the Northwest Institute of Eco-Environment and Resources, Chinese Academy of Sciences (protocol number: CAS201810082).

### 2.1. Experimental Animals and Design

Sheep feeding trials were carried out at the Gansu Gaolan Field Scientific Observation and Research Station for Agricultural Ecosystems of the Northwest Institute of Eco-Environment and Resources in the Chinese Academy of Sciences (36°13″ N, 103° 47″ E, 1780 m above sea level) [21].

Six 12-month-old rumen-fistulated Hu rams (56.2 ± 8.26 kg; mean ± SD) were penned (1.2 m × 0.6 m × 1.8 m) individually in a well-ventilated shed. The number of rams was based on the effect size index (d-value), which was calculated using the estimated standard deviations of the means of measured variables from previous similar studies. A sample size of 6 per group resulted in a d-value that was close to 0.5, which, according to Sullivan and Feinn, is a medium and acceptable effect size [22]. The rams were utilized in a 3 × 3 Latin square design, with three treatments, three periods, and 2 sheep in each treatment in each period. Treatments (*n* = 6 rams/treatment) consisted of three levels of FNT, namely, 0 g/d (control-CON), 16 g/d (N_16_), and 48 g/d (N_48_) on a dry matter basis, and each period lasted 23 days, with 14 days for adaptation and 9 days for data collection. The FNT used in this experiment was the residual small fruit, which was purchased from a herbal supplier.

The rams received 1500 g DM per day of a pelleted total mixed ration (Gansu Runmu Biological Engineering Co., Ltd., Jinchang, China): 800 g at 08:00 and 700 g at 18:00. The basal diet was formulated according to the requirements of NRC [23]. Feed ingredients and nutrition levels are presented in Table 1. The FNT was mixed with 100 g of the diet in the morning and was consumed in toto by the rams. Water and mineral salt blocks were freely available.

### 2.2. Sample Collection and Processing

Five ml of jugular-vein blood was collected using evacuated tubes (Shanghai Kehua Bio-Engineering Co., Ltd., Shanghai, China) from each ram before the morning feeding on the last day and centrifuged at 4 °C and 1200× *g* for 15 min. The serum was stored at −20 °C for analysis of biochemistry variables [24]. In addition, on the last day, at 12:00, approximately 100 mL of rumen content was collected via the fistula using a measuring cup and was strained through four layers of cheesecloth [25]. The pH was measured immediately after collection, using a portable pH meter (PHB-10, Shanghai Hongyi Instrument Limited, Shanghai, China), then the rumen fluid was stored in 10 mL centrifuge tubes at −80 °C for the determination of rumen fermentation variables, rumen enzyme concentrations, and rumen microbiota.

### 2.3. Serum Biochemical Variables

Serum biochemical variables were determined by Shanghai Bangyi Biological Technology (Shanghai, China). Total protein (TP, g/L), albumin (ALB, g/L), triglicerides (TG, mmol/L), glucose (GLU, mmol/L), blood urea nitrogen (BUN, mmol/L), and total cholesterol (TC, mmol/L) were measured using an automatic biochemistry analyzer (Mindray BS400, Shenzhen Mindray Bio-Medical Electronics Co., Ltd., Shenzhen, China). The immunoglobulin G (IgG, g/L) was calculated as the difference between TP and ALB. Non-esterified fatty acid (NEFA, µmol/L), β-hydroxybutyrate (BHBA, µmol/L), alkaline phosphatase (ALP, U/L), lactate dehydrogenase (LDH, U/L), pepsin (IU/L), lipase (IU/L), amylase (IU/mL), and cellulase (IU/L) were determined using enzyme-linked immunosorbent assay (ELISA) kits (Shanghai Bangyi Biological Technology, Shanghai, China). Antioxidant indices, including malondialdehyde (MDA, mmol/mL), glutathione peroxidase (GSH-Px, U/mL), superoxide dismutase (SOD, U/mL), total antioxidant capacity (T-AOC, U/mL), and catalase (CAT, U/mL) were determined using a microplate reader (Infinite F50, Tecan, Zurich, Switzerland).

### 2.4. Ruminal Fermentation Variables and Feed Composition Analysis

Five mL of rumen fluid were mixed with 1 mL of 250 g/L of metaphosphoric acid, then the volatile fatty acids (VFAs) were separated and quantified using gas chromatography/mass spectrometry (GC-MC) (7890B GC/5977B MSD, Agilent Technologies, Santa Clara, CA, USA) with a 30 m × 0.25 mm × 0.25 µm capillary column (AT-FFAP, Agilent Technologies, Santa Clara, CA, USA). Feed samples were dried to a constant weight in a forced air oven at 65°C, air-equilibrated for 12 h, then ground using a 1-millimeter screen. The DM of the feed was determined by drying for 24 h in a forced air oven at 105°C. The nitrogen (N) content (percentage of dry matter) in feed was determined by the Kjeldahl method, and crude protein content (percentage of dry matter) was calculated as N × 6.25 [26]. The ether extract (percentage of dry matter) was measured via Soxhlet extraction with diethyl ether for six hours.

### 2.5. Extraction of DNA and Polymerase Chain Reaction (PCR) Amplification

The total genomic DNA of the rumen bacteria was extracted using the E.Z.N.A.^®^ DNA kit (Omega Bio-Tek, Norcross, GA, USA) and following the manufacturer’s instructions. The quality of the extracted genomic DNA was tested by 1% agarose gel electrophoresis. The concentration and purity of the DNA were determined with a NanoDrop 2000 spectrophotometer (Thermo Scientific Company, Waltham, MA, USA) and stored at −80 °C. With the extracted DNA as the template, the V3–V4 variable region of the 16S rRNA gene of rumen bacteria was amplified by PCR using the upstream primer 338F (5’-ACTCCTACGGGAGGCAGCAG-3’) and the downstream primer 806R (5’-GGGACTACHVGGTWTCTAAT-3’) [27] carrying the barcode sequence, then the PCR products were recovered and purified.

### 2.6. Sequencing of the 16S rRNA Gene and Bioinformatics Analysis

The purified PCR products were sequenced on the Illumina Miseq PE300 platform (Shanghai Meiji Biomedical Technology Co., Ltd., Shanghai, China). FASTQ version 0.19.6 [28] software was used for quality control, FLASH version 1.2.11 [29] software was used for splicing, and UPARSE version 7.1 [30,31] software was used for operational taxonomic unit (OTU) clustering, according to 97% similarity and eliminated chimera. To minimize the effects of sequencing depth on alpha and beta diversity measurements, the sequence numbers of all samples were rarefied to 20,000 and the average sequence coverage (Good’s coverage) of each sample after plateauing was still up to 99.09%. The RDP classifier version 2.11 [32] was used for OTU taxonomic annotation on the Silva 16S rRNA gene database (v138). The confidence threshold was 0.7, and the community composition of each rumen fluid sample was counted at different species classification levels.

Mothur software [33] was used to calculate alpha diversity and Ace and Shannon indices, and the Wilcoxon rank-sum test tested for differences among groups. Constrained principal coordinate analysis (CPCoA), based on the Bray-Curtis distance algorithm, tested the dissimilarity of the microbial community structure between samples, and, combined with permutational multivariate analysis of variance analysis (PERMANOVA), tested whether the bacterial communities were distinct among groups. Linear discriminant analysis effect size (LEfSe) analysis [34] (LDA > 2, *p* < 0.05) was used to determine the bacterial groups, with significant differences in abundance from phylum to genus level among the different groups. Based on the Spearman correlation |r| > 0.6 and false discovery rate (FDR)-adjusted *p* < 0.05, species were selected for correlation analysis [35]. The sequencing data are stored in the sequence-reading archive (SRA) of the National Center for Biotechnology Information (NCBI) and can be obtained through the Bio Project number PRJNA880036.

### 2.7. Statistical Analysis

Initially, the Shapiro–Wilk test was used to test the data for normal distribution using SPSS 23.0 software (SPSS Inc., Chicago, IL, USA). The data that were distributed normally were analyzed by a one-way analysis of variance (ANOVA) [36], while the Kruskal–Wallis test analyzed data with non-normal distribution. Duncan’s test separated means when significance was detected. In addition, coefficients for unequally spaced contrasts were generated by the interactive matrix algebra procedure (IML) of SAS, then the linear and quadratic effects of the increasing FNT levels were assessed using orthogonal polynomial contrasts. The data are represented as means and the standard error of the mean (SEM). A level of *p* ≤ 0.05 was accepted as significant and 0.05 < *p* < 0.10 as tending to differ.

## 3. Results

### 3.1. Serum Biochemistry

Immunoglobulin G tended to increase (*p* = 0.08) with an increase in FNT intake and increased linearly (*p* = 0.03) with an increasing level of FNT (Table 2). Glucose did not differ (*p* = 0.14) among groups but displayed a quadratic curve (*p* = 0.05) with an increase in FNT. The FNT did not affect the serum concentrations of total protein, albumin, triglyceride, total cholesterol, and blood urea nitrogen.

### 3.2. Antioxidant Indices

There was no effect of FNT supplement on malondialdehyde (*p* = 0.80), glutathione peroxidase (*p* = 0.63), superoxide dismutase (*p* = 0.83), total antioxidant capacity (*p* = 0.72), and the concentration of catalase (*p* = 0.24; Table 3).

### 3.3. Serum Metabolites

The serum concentrations of β-hydroxybutyrate decreased linearly (*p* = 0.001; Table 4) with increasing FNT and were lower (*p* = 0.01) in the N_48_ group than in the CON group. Lactate dehydrogenase tended to decrease linearly (*p* = 0.07) with an increase in FNT intake. There was no difference in the serum concentrations of non-esterified fatty acids (*p* = 0.51), alkaline phosphatase (*p* = 0.67), and lactate dehydrogenase (*p* = 0.19) among the treatments.

### 3.4. Rumen Enzyme Concentrations

The rumen concentration of lipase decreased linearly (*p* = 0.04) with increasing FNT (Table 5). There was no difference in the rumen concentrations of pepsin (*p* = 0.91), amylase (*p* = 0.45), and cellulase (*p* = 0.59) among the groups.

### 3.5. Effects of the Fruit of Nitraria tangutorum (FNT) on Rumen pH and Concentrations of Volatile Fatty Acids in Hu Rams

Supplementary FNT did not affect the rumen pH (*p* = 0.26), the concentration of total volatile fatty acids (*p* = 0.80), and the molar proportions of acetate (*p* = 0.87), propionate (*p* = 0.87), butyrate (*p* = 0.55), and valerate (*p* = 0.30; Table 6).

### 3.6. Effects of the Fruit of Nitraria tangutorum (FNT) on the Rumen Bacterial Community Composition in Hu Rams

**Figure 1:**
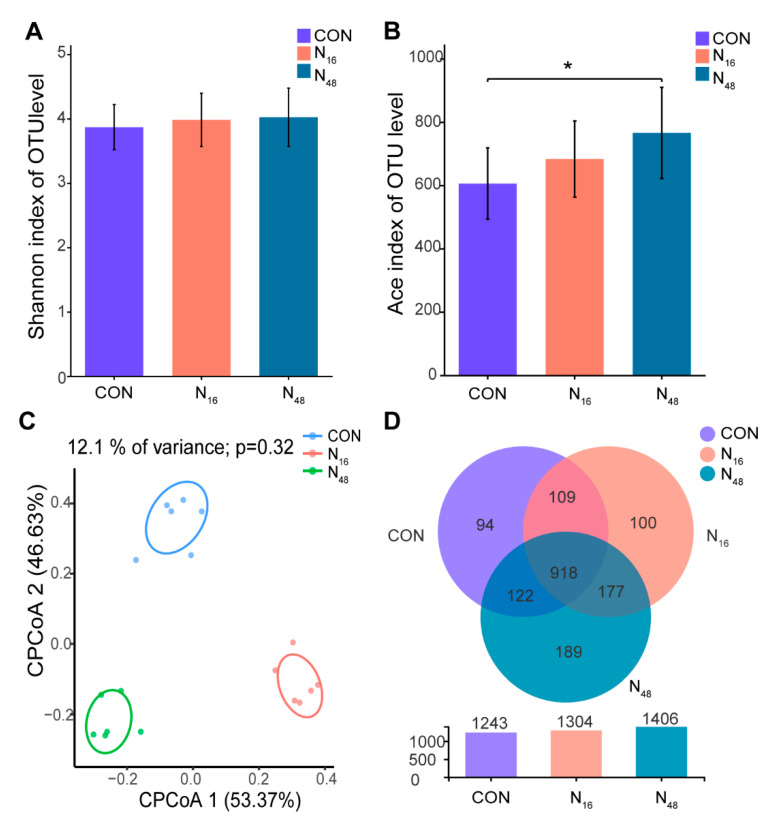
Effects of the fruit of *Nitraria tangutorum* (FNT) supplement on bacterial composition and structure in rumen fluid in Hu rams. (**A**) Shannon index of the OUT level; (**B**) Ace index of the OUT level; (**C**) CPCoA analysis of a bacterial community, based on OUT; (**D**) Venn diagram of OTUs in the three groups. * *p* < 0.05.

A total of 593,856 16S rRNA sequences were obtained from the sequencing of 18 microbial samples. The coverage rate of all samples was greater than 99%, and 1709 OTUs were obtained via sequence clustering and quality control (similarity > 97%). The curves reflecting the species number and diversity index tended to plateau (Figure S1 in supplementary materials), so sequencing depth accounted for most of the biomass in the sample. The Ace index, reflecting species richness, was greater (*p* = 0.045) in N_48_ than in CON (Figure 1B), while the Shannon index, reflecting species diversity, did not differ (*p* = 0.575) among treatments (Figure 1A). The rumen microbial community dissimilarity was determined by CPCoA analysis (Figure 1C). The two groups consuming FNT were separated from the CON group, but the difference was insignificant. The Venn diagram illustrated that there were 918 public core OTUs in the three groups, and 94, 100, and 189 unique OTUs in CON, N_16_, and N_48_, respectively (Figure 1D).

**Figure 2:**
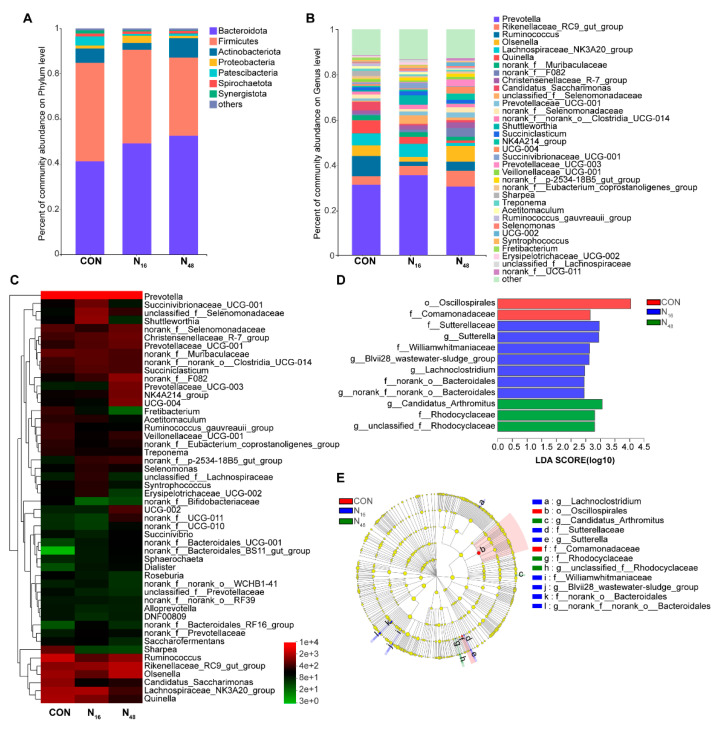
Effects of the fruit of *Nitraria tangutorum* (FNT) supplement on bacterial species composition and differential species in the rumen in Hu rams. (**A**) Changes in bacterial composition at the phylum level; (**B**) changes in bacterial composition at the genus level; (**C**) hierarchical cluster analysis of the relative abundances of rumen bacteria at the genus level; (**D**) analysis of the differential species, based on LEfSe; (**E**) Branch diagram of microbial evolution.

A total of 22 phyla and 334 genera of bacteria were identified in the rumen fluid. At the phylum level (with a relative abundance > 0.1%), Bacteroidota, Firmicutes, Actinobacteriota, Proteobacteria, Patescibacteria, Spirochaetota, and Synergistota were predominant across the 3 treatments (Figure 2A). At the genus level (with a relative abundance > 0.1%), *Prevotella*, *Rikenellaceae*_*RC9*_*gut*_*group*, *Ruminococcus*, *Olsenella*, *Lachnospiraceae*_*NK3A20*_*group*, *Quinella*, and *Christensenellaceae*_*R*-*7*_*group* were the most abundant (Figure 2B).

Hierarchical cluster analysis on the 50 bacteria genera with the highest relative abundances showed that *Prevotella* had the highest relative abundance in the three groups. *Quinella*, *Candidatus*, *Saccharimonas*, *Ruminococcus*, *Sharpea*, *Ruminococcus gauvreauii group*, *Fretibacterium*, and *Selenomona* were abundant in the CON group. *Shuttlewochaeta*, *Succinivibrionaceae*_*UCG*-*001*, *Succiniclasticum*, *Selenomonas*, *Syntrophococcus*, and *Erysipelotrichaceae*_*UCG*-*002* were most abundant in the N_16_ group. The N_48_ group was rich in *Rikenellaceae*_*RC9*_ *gut*_ *group*, *Olsenella*, *Prevotellaceae*_*UCG*-*003*, *NK4A214*_*group* and *UCG*-*004*. *Sphaerochaeta*, *Dialister*, *Christensenellaceae*_*R*-*7*_*group*, and *Prevotellaceae*_*UCG-001* increased with an increase in FNT level (Figure 2C).

LEfSe analysis was used to identify the difference in rumen bacteria among groups. The microbial groups with significant indigenous enrichment and influence on the different groups were counted, among which *Oscillospirales* and Comamonadaceae were identified in the CON group, genera including *Sutterella*, *Blvii28*_*wastewater*-*sludge*_*group*, *Lachnoclostridium*, and family including Sutterellaceae and Williamwhitmaniaceae were enriched significantly in the N_16_ group. *Candidatus* Arthromitus and Rhodocyclaceae, at the level of genus and family, respectively, were enriched significantly in the N_48_ group (Figure 2D,E).

### 3.7. Correlation Analysis between Differentially Abundant Bacteria and Serum Metabolite and Biochemical Variables

**Figure 3:**
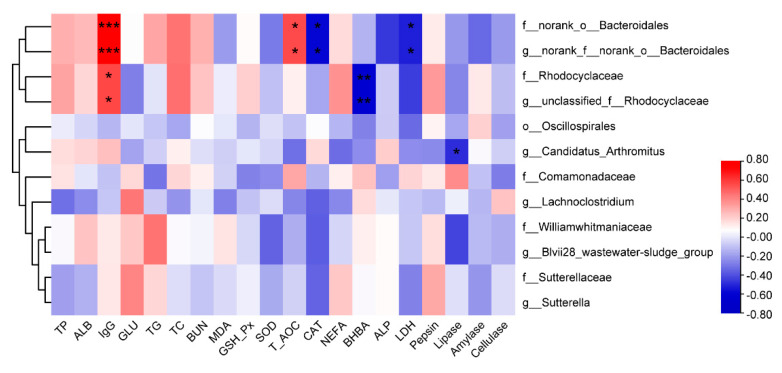
Correlation analysis between serum biochemical variables, rumen enzyme concentrations, and differential bacteria. The X and Y axes are environmental variables and species, respectively, and the r and *p*-values were obtained by Spearman analysis. The r values are displayed in different colors. The right image is the color interval of different r values. * 0.01 < *p* ≤ 0.05, ** 0.001 < *p* ≤ 0.01, *** *p* ≤ 0.001.

Correlation analysis revealed that Rhodocyclaceae was correlated positively (r = 0.632, *p* = 0.005) with serum IgG concentration and negatively (r = − 0.617, *p* = 0.007) with the serum concentration of BHBA. *Candidatus* Arthromitus was correlated negatively (r = − 0.485, *p* = 0.041) with lipase (Figure 3).

## 4. Discussion

The fruit of *Nitraria tangutorum* (FNT) is used widely as a food and medicine. This is the first study that has examined the effects of supplementary FNT on serum metabolite variables, rumen fermentation, and the rumen microbiota in sheep.

### 4.1. Effects of the Fruit of Nitraria Tangutorum (FNT) Supplement on Serum Biochemistry Variables in Hu Rams

Serum biochemical variables are used to indicate the condition, metabolism, and health status of the animal [37]. For example, serum concentrations of BUN, TP, ALB, and IgG are key indicators reflecting animal protein metabolism [38]. Different dietary treatments can affect the level of IgG in ruminant serum [39,40]. In the current study, IgG increased with an increase in the level of FNT intake. The IgG affects the growth of lambs [40,41] and their immune functions [39], which may be related to the effective bioactive components, as FNT is rich in polysaccharides [13], flavonoids, alkaloids [20], and anthocyanins [42]. Natural polysaccharides not only enhance immunity, but also regulate it by acting directly on the immune cells and targeting the gut microbiota [43]. The FNT did not affect the serum concentrations of BUN, TP, and ALB levels. The rams in the present study were mature and were not growing. A study on growing lambs is warranted to examine the effect of FNT on protein metabolism and utilization. The above results indicated that the FNT supplementation in this study did not affect the metabolism and utilization of dietary protein, at least in mature Hu rams, but had a tendency to improve immunity.

Blood lipids are essential substances for basal metabolism in the body and can reflect lipid metabolism in animals. TG is affected mainly by dietary intake and nutrient levels and is an important form of energy storage for ruminants. Serum concentrations reflect fat metabolism, and an increase in concentration could indicate malnutrition or stress. There was no difference among treatments, probably because the animals were not under nutritional stress. TC is synthesized mainly by the liver and is essential for cell-membrane ACTH, bile acid, vitamin D, and hormones [44,45]. The concentrations for the rams in the present study did not differ among treatments and were similar to the 3.69 to 6.37 U/L reported for dairy cows [46] and the 4.15 mmol/L reported for water buffaloes (*Bubalus bubalis*) [47].

In this study, FNT did not affect the serum concentrations of MDA, GSH-Px, T-AOC, CAT, and SOD, indicating that dietary FNT supplementation did not affect the antioxidant capacity of the rams. However, FNT did affect the antioxidant capacity in humans [10], while proanthocyanins reduced oxidative stress in rats [16]. This would suggest that sheep respond differently to FNT than humans and rats, or that the dose offered to the sheep in the present study was insufficient. There is some support for the latter, as the serum concentrations of GSH-Px increased numerically with an increase in FNT intake, although the differences were not significant. The increase may be due to the antioxidant effect of FNT [48,49]; however, more evidence is needed to establish the anti-oxidative effect of FNT on sheep.

Serum metabolites are commonly used to assess the nutritional status of livestock [50]. In the current study, BHBA values decreased with increasing FNT levels, but FNT treatment did not affect the serum concentrations of NEFA, GLU, ALP, and LDH. BHBA is commonly used to detect ketosis, and a concentration of 1 mmol/L is considered a subclinical level in cows [51]. Although there was a decrease in the serum concentration of BHBA with an increase in FNT intake, all groups were below the subclinical level. FNT contains abundant polysaccharides [13] that can be used to provide energy for the animal [52]. Therefore, FNT supplementation in the diet can reduce the mobilization of adipose tissue and liver glycogen, and also reduce the serum concentration of BHBA. This could explain the decrease in BHBA concentration with an increase in FNT level. The serum NEFA concentration reflects fat metabolism, and the unchanged concentration may be related to the absence of changes in TC. GLU is regulated mainly by the liver [53], and a serum concentration of less than 2.5 mmol/L, indicates metabolic disorders [54]. The concentration of GLU in this study was between 4.33 and 4.97 mmol/L, which were all above this threshold, indicating that the sheep were well nourished. The serum concentration of LDH decreased linearly with the increasing FNT intake in the current study. LDH is one of the main enzymes involved in gluconeogenesis and is mainly involved in energy metabolism [55]. The concentration of LDH in the present study for all groups was in the normal range of 88–487 U/L commonly reported for sheep [56].

### 4.2. Effects of the Fruit of Nitraria Tangutorum (FNT) Supplement on Rumen pH and Rumen Fermentation in Hu Rams

Rumen pH and VFAs reflect the function and stability of the internal rumen environment. In the present study, the rumen pH ranged between 5.60 and 5.94 and did not differ among treatments. The pH values were slightly lower than the optimal range of 6.2 to 7.2 for fibrolytic bacteria [57]. This may be related to the ratio of concentrate to roughage in the diet for this study, as high-grain diets can lower rumen pH [58,59]. In ruminants, VFAs provide approximately 70% of the energy demands of the ruminant [60]. In dairy cows, the active polysaccharides regulated rumen fermentation and increased the total VFA production [61]. However, in spite of the polysaccharides in FNT, the concentration of ruminal VFAs did not differ among groups in the present study.

### 4.3. Effects of the Fruit of Nitraria Tangutorum (FNT) Supplement on Rumen Microorganisms in Hu Rams

The diversity of the rumen microbial community is affected largely by diet [62]. The Ace index of alpha diversity increased with an increase in FNT, which indicates that the FNT increased the microbial richness in the rumen. Greater microbial diversity in the mammalian gastrointestinal tract usually means stronger metabolic capacity and stability [63]. Its unique community structure and metabolites are essential for regulating host metabolism, growth and development, and immune regulation [64].

In the current study, Firmicutes and Bacteroidetes were the two most abundant bacterial phyla in the rumen, which is consistent with other studies on ruminants [40,65,66,67]. The relative abundance of Bacteroidetes, which is important in the degradation of polysaccharides such as cellulose, hemicellulose, and pectin [68,69], increased with the addition of FNT to the diet. FNT contains a large number of bioactive components, including polysaccharides, which are beneficial to rumen microorganisms [70,71] that enhance the relative abundance of Bacteroidetes [72].

At the genus level, the relative abundances of *Rikenellaceae*_*RC9*_*gut*_*group* and *Christensenellaceae*_*R*-*7*_*group* increased, whereas *Quinella* and *Ruminococcus* decreased with supplementary dietary FNT. *Rikenellaceae*_*RC9*_*gut*_*group* belongs to the Bacteroidetes that promote lipid metabolism [73] and carbohydrate and protein fermentation [74]. Recent studies reported that the *Christensenellaceae*_*R*-*7*_*group* exists widely in the intestine and mucosa of the host, is essential to health, and is involved in amino acid and lipid metabolism [75]. The relative abundance of the conditional pathogenic bacteria, *Quinella* [76,77,78], decreased with an increase in FNT, which indicates that FNT has the potential to improve immunity. The active ingredient polysaccharides can reduce pathogenic bacteria, promote the growth and reproduction of beneficial ruminal bacteria, and reduce the consumption of carbohydrates and other nutrients by pathogenic bacteria [7].

We further analyzed the differential bacteria in each group and found that supplementary FNT increased the relative abundance of beneficial bacteria. *Oscillospirales* and Comamonadaceae were enriched significantly in the CON group, along with *Sutterella* and *Lachnoclostridium* in the N_16_ group and *Candidatus* Arthromitus and Rhodocyclaceae in the N_48_ group. *Sutterella* is an anaerobic or microaerobic Gram-negative bacterium [79] that acts as an immunoregulator in the human gastrointestinal tract [80,81]. The increase in the relative abundance of *Lachnoclostridium* may be due to the utilization of the polysaccharides contained in FNT. *Lachnoclostridium* is involved in the degradation of polysaccharides and in the synthesis of amino acids in the rumen [82]. It was also reported that *Lachnoclostridium* can improve the intestinal barrier function of mice [83] and had a positive impact on the colon development of dairy cows [84]. In addition, the intake of FNT increased the relative abundance of *Candidatus* Arthromitus, commonly referred to as segmented filamentous bacteria (SFB). It is a symbiotic bacterium that regulates the maturation of the host immune system, stimulates the differentiation of Th17 cells, and promotes the secretion of intestinal surface immunoglobulin A (sIgA) [33,85]. Therefore, the increase in *Candidatus* Arthromitus may be related to the immunomodulation of FNT. Correlation analysis revealed that *Candidatus* Arthromitus was correlated inversely with lipase, as there was a linear decrease in lipase with an increase in the relative abundance of *Candidatus* Arthromitus. The denitrifying bacteria, Comamonadaceae, was enriched in the CON group, while Rhodocyclaceae was enriched significantly in the N_48_ group. Rhodocyclaceae in the rumen of lambs is correlated positively with rumen development, while Comamonadaceae is correlated negatively with host growth performance and rumen development [86]. In addition, the Spearman correlation results revealed that Rhodocyclaceae was correlated positively with IgG. These results indicate that FNT has beneficial effects on the rumen and immune function in sheep. Other medicinal plants are reputed to possess immunoregulatory effects [87]. Interestingly, the enriched bacteria species differed between the two levels of FNT consumption. The reason for this is unknown and warrants further research.

## 5. Conclusions

Supplementary FNT altered the immune-related indicators in the serum and increased the relative abundances of beneficial bacteria, such as *Lachnoclostridium*, Rhodocyclaceae, and *Candidatus* Arthromitus in the rumen of Hu rams. In addition, FNT influenced the serum concentrations of BHBA and LDH along with the rumen concentration of lipase, which are all involved in energy metabolism. Further studies are warranted on the effect of supplementing FNT on fattening sheep and in determining the optimal level that should be supplemented in the feed.

## Figures and Tables

**Table 1:** Ingredients and chemical compositions of the diet used in the study.

Items	Basal Diet
Corn straw	25.00
Corn bran	10.70
Corn grain	22.50
Barley	15.00
Molasses	4.00
Corn germ meal	15.00
Soybean meal	5.70
Limestone	1.10
Salt	0.50
Premix ^A^	0.50
Chemical composition	
DM ^B^, %	87.87
Crude protein, % DM	11.00
Ether extract, % DM	1.69
P, % DM	0.26
Ca, % DM	0.90
Metabolic energy ^C^, MJ/kg DM	9.60

^A^ The premix provided the following component per kg: Fe, 2000 PPM; Zn, 8000 PPM; Cu, 500 PPM; Mn, 4000 PPM; Se, 80 PPM; Co, 80 PPM; I, 70 PPM; VA, 1200 KIU; VD, 300 KIU; VE, 10,000 IU. ^B^ DM, dry matter. ^C^ Calculated as N × 6.25.

**Table 2:** Effects of the fruit from *Nitraria tangutorum* (FNT) supplement on serum biochemical variables in Hu rams.

Items	Groups			SEM ^A^	*p*-Value		
	CON	N_16_	N_48_		Treatment	Linear	Quadratic
Total protein, g/L	82.3	76.2	84.4	2.53	0.42	0.75	0.21
Albumin, g/L	46.7	45.6	49.8	1.67	0.60	0.47	0.48
Immunoglobulin G, g/L	22.3	30.6	34.6	2.32	0.08	0.03	0.63
Glucose, mmol/L	4.43	4.97	4.33	0.14	0.14	0.76	0.05
Triglyceride, mmol/L	1.51	1.70	1.60	0.07	0.63	0.68	0.39
Total cholesterol, mmol/L	4.56	4.98	5.51	0.20	0.39	0.20	0.67
Blood urea nitrogen, mmol/L	4.82	5.08	5.32	0.17	0.52	0.26	0.97

CON, 0 g FNT/d; N_16_, 16 g FNT/d; N_48_, 48 g FNT/d. ^A^ SEM, standard error of the mean.

**Table 3:** Effects of the fruit of *Nitraria tangutorum* (FNT) supplement on serum antioxidant indices in Hu rams.

Items	Groups			SEM ^A^	*p*-Value		
	CON	N_16_	N_48_		Treatment	Linear	Quadratic
Malondialdehyde, mmol/mL	7.78	8.26	7.69	0.36	0.80	0.92	0.52
Glutathione peroxidase, U/mL	817	849	900	34.0	0.63	0.35	0.90
Superoxide dismutase, U/mL	64.5	62.8	59.0	3.55	0.83	0.56	0.90
Total antioxidant capacity, U/mL	16.2	16.7	15.6	0.52	0.72	0.63	0.52
The contents of catalase, U/mL	184	141	155	10.5	0.24	0.26	0.21

CON, 0 g FNT/d; N_16_, 16 g FNT/d; N_48_, 48 g FNT/d. ^A^ SEM, standard error of the mean.

**Table 4:** Effects of the fruit of *Nitraria tangutorum* (FNT) supplement on serum metabolites in Hu rams.

Items	Groups			SEM ^A^	*p*-Value		
	CON	N_16_	N_48_		Treatment	Linear	Quadratic
Non-esterified fatty acid, µmol/L	686	736	762	26.2	0.51	0.26	0.84
β-hydroxybutyrate, µmol/L	309 ^a^	294 ^a^	235 ^b^	11.4	0.01	0.001	0.26
Alkaline phosphatase, U/L	95.6	86.8	93.4	3.97	0.67	0.83	0.40
Lactate dehydrogenase, U/L	171	159	145	5.9	0.11	0.07	0.93

CON, 0 g FNT/d; N_16_, 16 g FNT/d; N_48_, 48 g FNT/d. ^A^ SEM, standard error of the mean. ^a,b^ Means with different letters in the same row differ from each other (*p* < 0.05).

**Table 5:** Effects of the fruit of *Nitraria tangutorum* (FNT) supplement on rumen enzyme concentrations in Hu rams.

Items	Groups			SEM ^A^	*p*-Value		
	CON	N_16_	N_48_		Treatment	Linear	Quadratic
Pepsin, IU/L	128	129	133	4.5	0.91	0.68	0.90
Lipase, IU/L	184	165	137	9.2	0.10	0.04	0.79
Amylase, IU/mL	77.1	69.0	79.1	3.30	0.45	0.82	0.22
Cellulase, IU/L	267	301	283	12.9	0.59	0.63	0.37

CON, 0 g FNT/d; N_16_, 16 g FNT/d; N_48_, 48 g FNT/d. ^A^ SEM, standard error of the mean.

**Table 6:** Effects of the fruit of *Nitraria tangutorum* (FNT) supplement on ruminal pH and fermentation variables in Hu rams.

Items	Groups			SEM ^A^	*p*-Value		
	CON	N_16_	N_48_		Treatment	Linear	Quadratic
pH	5.69	5.60	5.94	0.12	0.26	0.41	0.40
Total VFA, mmol/L	81.5	95.6	80.8	9.82	0.80	0.78	0.64
Acetate, mmol/L	49.2	57.1	53.3	5.53	0.87	0.97	0.60
Propionate, mmol/L	25.1	29.4	24.7	3.81	0.87	0.65	0.34
Butyrate, mmol/L	6.50	7.86	5.43	0.90	0.55	0.66	0.14
Valerate, mmol/L	0.54	1.09	0.71	0.15	0.25	0.98	0.51

CON, 0 g FNT/d; N_16_, 16 g FNT/d; N_48_, 48 g FNT/d. VFA, volatile fatty acid. ^A^ SEM, standard error of the mean.

## Data Availability

The data presented in the study are available on request from the corresponding author.

## Supplementary Materials

The following supporting information can be downloaded at: https://www.mdpi.com/article/10.3390/ani12223211/s1, Figure S1: Effect of FNT addition on rarefaction curves in the rumen fluid of Hu rams.
