# Supplementary material for: Dietary Supplementation of Fruit from Nitraria tangutorum Improved Immunity and Abundance of Beneficial Ruminal Bacteria in Hu Sheep
Source: Animals (Basel). 2022 Nov 19;12(22):3211. doi: 10.3390/ani12223211 (PMC9686964; doi:10.3390/ani12223211)
Supplement: Supplementary file 1 [file animals-12-03211-s001.zip › animals-2006677-supplementary.pdf]

animals

MDPI

**Figure S1**

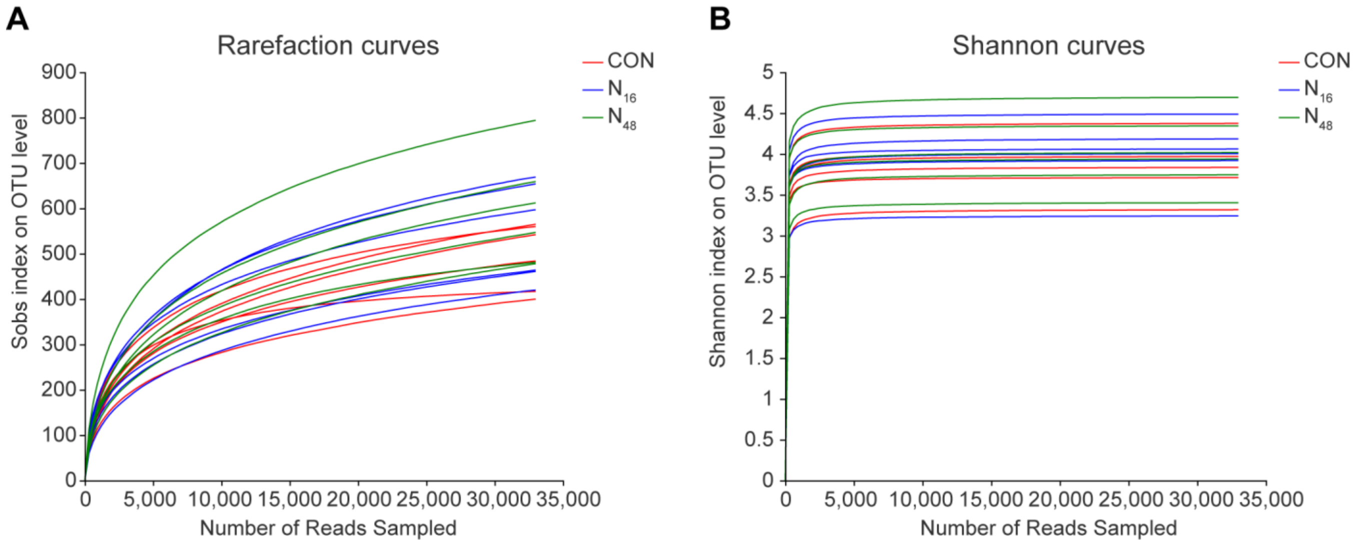


**Figure S1.** Effects of *Nitraria tangutorum* (FNT) addition on rarefaction curves in rumen fluid in Hu rams.
